# Associations between homocysteine metabolism related SNPs and carotid intima-media thickness: a Chinese sib pair study

**DOI:** 10.1007/s11239-016-1449-x

**Published:** 2016-11-07

**Authors:** Kexin Sun, Jing Song, Kuo Liu, Kai Fang, Ling Wang, Xueyin Wang, Jing Li, Xun Tang, Yiqun Wu, Xueying Qin, Tao Wu, Pei Gao, Dafang Chen, Yonghua Hu

**Affiliations:** 1 Department of Epidemiology and Biostatistics, Peking University Health Science Center, 38 Xueyuan Road, Beijing, 100191 China; 2 Department of Epidemiology and Biostatistics, Capital Medical University, 10 You’anmenwai Xitoutiao, Beijing, 100069 China; 3 Beijing Center for Disease Prevention and Control, No.16 He Pingli Middle Street, Dongcheng District, Beijing, 100013 China; 4 Pingshan New District Center for Disease Control and Prevention, Shenzhen, 518118 Guangdong China

**Keywords:** Homocysteine, Single nucleotide polymorphism, Carotid intima-media thickness, Atherosclerosis, Sib pair, Gene–gene interaction

## Abstract

Carotid intima-media thickness (CIMT) is a good surrogate for atherosclerosis. Hyperhomocysteinemia is an independent risk factor for cardiovascular diseases. We aim to investigate the relationships between homocysteine (Hcy) related biochemical indexes and CIMT, the associations between Hcy related SNPs and CIMT, as well as the potential gene–gene interactions. The present study recruited full siblings (186 eligible families with 424 individuals) with no history of cardiovascular events from a rural area of Beijing. We examined CIMT, intima-media thickness for common carotid artery (CCA-IMT) and carotid bifurcation, tested plasma levels for Hcy, vitamin B6 (VB6), vitamin B12 (VB12) and folic acid (FA), and genotyped 9 SNPs on MTHFR, MTR, MTRR, BHMT, SHMT1, CBS genes. Associations between SNPs and biochemical indexes and CIMT indexes were analyzed using family-based association test analysis. We used multi-level mixed-effects regression model to verify SNP-CIMT associations and to explore the potential gene–gene interactions. VB6, VB12 and FA were negatively correlated with CIMT indexes (*p* < 0.05). rs2851391 T allele was associated with decreased plasma VB12 levels (*p* = 0.036). In FABT, CBS rs2851391 was significantly associated with CCA-IMT (*p* = 0.021) and CIMT (*p* = 0.019). In multi-level mixed-effects regression model, CBS rs2851391 was positively significantly associated with CCA-IMT (Coef = 0.032, se = 0.009, raw *p* < 0.001) after Bonferoni correction (corrected α = 0.0056). Gene–gene interactions were found between CBS rs2851391 and BHMT rs10037045 for CCA-IMT (*p* = 0.011), as well as between CBS rs2851391 and MTR rs1805087 for CCA-IMT (*p* = 0.007) and CIMT (*p* = 0.022). Significant associations are found between Hcy metabolism related genetic polymorphisms, biochemical indexes and CIMT indexes. There are complex interactions between genetic polymorphisms for CCA-IMT and CIMT.

## Introduction

Carotid intima-media thickness (CIMT), a technique to monitor carotid artery wall alterations using ultrasonography, is a noninvasive measurement of preclinical atherosclerosis [1]. It has often been considered as a surrogate of early atherosclerosis and is widely used for cardiovascular risk stratification [2].

Hyperhomocysteinemia is an independent risk factor for arteriosclerotic vascular diseases [3, 4]. However, the association between elevated plasma homocysteine (Hcy) and CIMT remains controversial [5]. This prompts the need for genetic association studies to investigate the involvement of Hcy in early stage of atherogenesis.

Hcy can be influenced by genetic factors. Altered functioning of catalytic enzymes in Hcy metabolic pathways caused by gene mutations may lead to inhibition of certain pathways and elevation of plasma Hcy level [6, 7]. Methylenetetrahydrofolate reductase (MTHFR), methionine synthase (MTR), methionines synthase reductase (MTRR), betaine-homocysteine methyltransferase (BHMT) are enzymes in Hcy remethylation pathway. Cystathionine-β-synthase (CBS) is the key enzyme in Hcy transsulfuration pathway. Serine hydroxymethyltransferase 1 (SHMT1) sequesters 5-methyltetrahydrofolate, a co-substrate for homocysteine remethylation, thus to inhibit Hcy metabolism through methionine synthesis pathway. Genetic association studies between encoding genes of these enzymes and cardiovascular studies are previously studied [8, 9].

Different enzymes in Hcy metabolic pathways interact with each other. Complex interactions between variations of corresponding genes, especially interactions between functional polymorphisms, can contribute to Hcy metabolic disorder and subsequent diseases. Interactions between MTHFR 677 C>T (rs1801133) and CBS 844ins68 [10–12], MTHFR 677 C>T and MTRR 66 A>G (rs1801394) [13] were found with regard to Hcy. Same interactions were shown to elevate neural tube defects risk [14]. However, the interactive effects of Hcy related SNPs on CIMT were not fully explored.

To investigate the associations between Hcy metabolism related SNPs, Hcy related biochemical indexes and CIMT, we conducted a sib pair study in Chinese population and genotyped 9 cardiovascular diseases associated SNPs on 6 Hcy metabolism related genes using candidate gene approach. The aims of the present study are: (1) to explore associations between SNPs and Hcy, vitamin B6 (VB6), vitamin B12 (VB12), folic acid (FA) levels; (2) to test the associations between SNPs and CIMT indexes; (3) to explore the potential gene–gene interactions for CIMT indexes.

## Methods

### Study design

The current study nested in Fangshan Family-based Ischemic Stroke Study In China (FISSIC) program. FISSIC has been described in details elsewhere [15]. In brief, it is an ongoing family-based genetic epidemiological study starting from June 2005. We recruited Northern Chinese Han pedigrees from communities in Fangshan District, Beijing, China. The inclusion criteria for this study were: (1) >40 years old; (2) full siblings; (3) with no medical history of cardiovascular events (including angina pectoris, myocardial infarction, sudden cardiac death, ischemic stroke, hemorrhagic stroke, transient ischemic attack, or other cardiovascular diseases) or antihyperlipidemia treatment at base line. The exclusion criteria were: (1) receiving extra vitamin B or folic acid supplements regularly; (2) receiving drugs which can influence Hcy levels (methotrexated, isoniazide, azathioprine, thiazide diuretics); (3) incomplete genotyping data or other important parameters.

The present study included 186 eligible families with 424 individuals. Written informed consent was obtained from every participant. This project was approved by the Ethics Committee of Peking University Health Science Center, Beijing, China.

### Carotid intima-media thickness measurement

Every participant was scanned by one of the two trained ultrasonographers with high-resolution B-mode ultrasound system (Acuson Inc., Mountain View, CA, USA), using 7.5- to 10.0-MHz linear transducers according to study protocol.

Bilateral carotid arteries were scanned from its origin to bifurcation in transverse plane through an anterolateral approach. Longitudinal views were obtained in three segments: (1) distal common carotid artery (CCA) (2 cm proximal to the dilatation of the carotid bulb); (2) proximal CCA (1 cm proximal to the dilatation of the carotid bulb); (3) bifurcation (1 cm proximal to the flow divider). The B-mode ultrasound images were recorded for at least 6 cardiac cycles when the intimal–lumen interface of far wall was displayed as a sharp-edged continuous straight line.

IMT is a double-line pattern represented as the area between the artery luminal edge and media-adventitia interface [1]. IMT was analyzed by 3 qualified readers blinded to participants’ information using semi-automatic edge-detection software (Vasular Research Tool Carotid Analyzer, Medical Imaging Applications, Coralville, IA) in regions free of plaques. The mean IMT of far wall was recorded and was involved in further calculation. In each segment, we measured IMT both in diastole and systole frames and averaged the two values. The common carotid artery IMT (CCA-IMT) was the mean of 4 values obtained in bilateral distal and proximal CCA segments. The bifurcation IMT (Bif-IMT) was the mean of values in bilateral bifurcation segments. Carotid IMT (CIMT) was the mean of IMT in all segments.

To control the quality of IMT measurement, the reliability of scanning and reading procedures was evaluated. The intra-class correlation coefficients for two sonographers were both over 0.93, while the inter-class correlation coefficient was 0.90. The intra-reader correlation coefficients were 0.95 and 0.90 for CCA-IMT and Bif-IMT. The inter-reader correlation coefficients were 0.88 and 0.86 for CCA-IMT and Bif-IMT.

### Anthropometric measurements and biochemical analyses

The details of anthropometric measurements [including height (m), weight (kg), body mass index (BMI, kg/m^2^), systolic blood pressures (SBP, mmHg), diastolic blood pressures (DBP, mmHg), smoking status, drinking status] and biochemical analyses [fasting blood glucose (FBG, mmol/L), total cholesterol (TC, mmol/L), total triglycerides (TG, mmol/L), high density lipoprotein cholesterol (HDL-C, mmol/L), low density lipoprotein cholesterol (LDL-C, mmol/L)] were described elsewhere [15]. Medical history of cardiovascular events was collected through local surveillance system consisting of community medical centers, township hospitals and district hospitals. Current and ex-smokers were both treated as smokers during the analysis, as well as drinkers. Hypertension was defined as a diagnosis of hypertension, antihypertensive therapy, SBP ≥ 140 mmHg or DBP ≥ 90 mmHg during examination. Diabetes was defined as a diagnosis of diabetes, antidiabetic therapy, or FBG ≥ 7.0 mmol/L.

### Assessment of homocysteine, vitamin B6, vitamin B12 and folic acid levels

We collected fasting venous blood sample from every participant. Hcy (μmol/L) (Mindray, Shenzhen, China), VB6 (pg/ml), VB12 (pg/ml), FA (pg/ml) (Cusabio, Wuhan, China) and plasminogen activator inhibitor-1 (PAI-1, pg/ml) (Boster, Wuhan, China) concentrations were assessed by commercially available ELISA kits using TECAN GENios Plus enzyme-linked immunosorbent assay reader (TECAN, Grödig, Austria).

### SNPs selection and genetic analysis

We investigated 9 single nucleotide polymorphisms (SNPs) in 6 well-studied candidate genes (MTHFR, MTR, MTRR, BHMT, SHMT1, CBS) involved in homocysteine metabolism. SNPs previously reported to be associated with homocysteine levels or cardiac-cerebral vascular diseases were selected.

DNA was extracted from blood samples. We performed DNA genotyping using MassARRAY iPLEX platform (Sequenom Inc, San Diego, California, USA) following the manufacturer’s protocol. We assessed SNP genotypes by MassARRAY Typer Analyzer version 4.0. The call rates for 9 SNPs were all above 99.0%. To verify reproducibility, a randomly chosen subgroup of 5% samples went through repeat analysis. The results of these duplicated samples were 100% consistent.

### Statistical analysis

We analyzed TG, Hcy, VB6, VB12, FA, PAI-1 data on natural logarithmic scale owing to their skewed distribution. Continuous variables were described as the mean ± standard deviation and Student’s t test was adopted to compare means across gender groups. Categorical variables were described as frequency and proportion, and Pearson’s χ^2^ test was used for comparisons between gender groups.

We investigated correlations between Hcy related biochemical indexes and CIMT indexes, and the mean levels of these biochemical indexes in every SNP genotype groups.

We estimated Hardy–Weinberg equilibrium (HWE) for SNPs in all participants and found no violation (Table 4). When investigated under additive genetic models, genotypes with 0, 1 or 2 risk allele copies were coded as 0, 1 and 2 respectively. When investigated under dominant genetic models, genotypes with 0 risk allele copies were coded as 0, while genotypes with 1 or 2 risk allele copies were coded as 1.

Family-based association test in genetic analyses (FBAT) is a method testing for linkage as well as association using familial data. It is built on the original transmission disequilibrium test (TDT) method and is suitable for extended pedigrees such as sib pairs [16]. We used FBAT to estimate associations between SNPs and Hcy related biochemical indexes, as well as CIMT indexes under additive models.

To replicate the significant findings in FBAT, we employed multi-level mixed-effects regression model, which can accommodate correlation between full siblings. Bonferoni-corrected α value was used as the level of significance. SNPs with *p* < 0.05/9 = 0.0056 were considered significant.

We estimated gene–gene interactions between SNPs significantly associated with CIMT indexes and other SNPs under dominant model by adding multiplicative terms in multi-level mixed-effects regression models.

FBAT analysis was conducted using PBAT software (v3.6). Other statistical analyses were performed by STATA (version 13, Stata Corporation, Texas, USA).

## Results

### Basic characteristics of participants and sib pairs

The correlations between CCA-IMT and Bif-IMT were 0.754 and 0.786 in men and women respectively. Compared with males, females had lower rates of smoking and drinking. Females also had lower SBP and DBP levels, as well as higher HDL-C levels. The CIMT indexes for females were thinner. The prevalence of hypertension in females was lower than in males (Table 1).

**Table 1:** Basic anthropometric characteristics of participants

	Total	Male	Female	p*
*n*	424	237 (50.2)	187 (49.8)	–
Age	53.9 ± 9.3	54.4 ± 9.5	53.2 ± 9.0	0.231
Smoking	185 (43.6)	164 (69.2)	21 (11.2)	<0.001^^^
Drinking	131 (30.9)	117 (49.4)	14 (7.5)	<0.001^^^
BMI	26.0 ± 3.5	25.9 ± 3.4	26.0 ± 3.7	0.733
SBP	136.4 ± 20.1	139.2 ± 19.0	133.6 ± 20.8	<0.001^^^
DBP	83.3 ± 11.8	85.5 ± 12.0	81.2 ± 11.2	<0.001^^^
FBG	5.69 ± 2.21	5.81 ± 2.24	5.57 ± 2.18	0.097
TC	3.76 ± 1.04	3.75 ± 1.01	3.77 ± 1.06	0.770
TG	0.37 ± 0.63	0.41 ± 0.64	0.31 ± 0.61	0.102
HDL-C	1.20 ± 0.43	1.17 ± 0.40	1.23 ± 0.45	0.046^^^
LDL-C	2.64 ± 0.67	2.65 ± 0.71	2.62 ± 0.63	0.661
Hcy	2.63 ± 0.46	2.67 ± 0.42	2.59 ± 0.50	0.182
VB6	9.09 ± 0.58	9.05 ± 0.58	9.13 ± 0.59	0.314
VB12	6.38 ± 0.60	6.38 ± 0.59	6.39 ± 0.60	0.867
FA	8.85 ± 0.51	8.85 ± 0.50	8.85 ± 0.52	0.966
PAI-1	11.58 ± 0.76	11.63 ± 0.67	11.52 ± 0.86	0.278
CCA-IMT	0.75 ± 0.01	0.77 ± 0.17	0.72 ± 0.01	0.002^^^
Bif-IMT	0.80 ± 0.18	0.82 ± 0.19	0.77 ± 0.15	0.004^^^
CIMT	0.76 ± 0.16	0.78 ± 0.17	0.73 ± 0.14	0.001^^^
Diabetes mellitus	73 (17.2)	42 (17.7)	31 (16.6)	0.757
Hypertension	250 (59.0)	156 (65.8)	94 (50.3)	0.003^^^

Continuous variables were described as mean ± standard deviation. Categorical variables were described as frequency (proportion)

*BMI* body mass index, kg/m^2^; *SBP* systolic blood pressure, mmHg; *DBP* diastolic blood pressure, mmHg; *FBG* fast blood glucose, mmol/L; *TC* total cholesterol, mmol/L; *TG* total triglycerides, mmol/L; *HDL-C* high-density lipoprotein cholesterol, mmol/L; *LDL-C* low-density lipoprotein cholesterol, mmol/L; *Hcy* homocysteine, μmol/L; *VB6* vitamin B6, pg/ml; *VB12* vitamin B12, pg/ml; *FA* folic acid, pg/ml; *PAI-1* plasminogen activator inhibitor-1, pg/ml; *CCA-IMT* common carotid artery intima-media thickness, mm; *Bif-IMT* bifurcation artery intima-media thickness, mm; *CIMT* carotid artery intima-media thickness, mm. TG, Hcy, VB6, VB12, FA, PAI-1 were analyzed on natural logarithmic scale

*p values for comparisons across different gender groups, ^^^ *p* < 0.05

### Correlations between biochemical indexes and carotid intima-media thickness

VB6 (CCA-IMT: Corr = −0.283, *p* < 0.001; Bif-IMT: Corr = −0.234, *p* < 0.001; CIMT: Corr = −0.277, *p* < 0.001), VB12 (CCA-IMT: Corr = −0.194, *p* = 0.002; Bif-IMT: Corr = −0.173, *p* = 0.007; CIMT: Corr = −0.198, *p* = 0.002) and FA (CCA-IMT: Corr = −0.371, *p* < 0.001; Bif-IMT: Corr = −0.280, *p* < 0.001; CIMT: Corr = −0.363, *p* < 0.001); were negatively correlated with CIMT indexes. No correlations were found between Hcy, PAI-1 and CIMT indexes (Table 2).

**Table 2:** Correlations between biochemical indexes and carotid intima-media thickness

Variables	CCA-IMT		Bif-IMT		CIMT	
	Corr	*p*	Corr	*p*	Corr	*p*
Hcy	0.092	0.153	0.055	0.396	0.030	0.652
VB6	−0.283	<0.001^^^	−0.234	<0.001^^^	−0.277	<0.001^^^
VB12	−0.194	0.002^^^	−0.173	0.007^^^	−0.198	0.002^^^
FA	−0.371	<0.001^^^	−0.280	<0.001^^^	−0.363	<0.001^^^
PAI-1	−0.011	0.861	0.012	0.848	−0.002	0.973

*Hcy* homocysteine, *VB6* vitamin B6, *VB12* vitamin B12, *FA* folic acid, *PAI-1* plasminogen activator inhibitor-1, *CCA-IMT* common carotid artery intima-media thickness, *Bif-IMT* bifurcation artery intima-media thickness, *CIMT* carotid artery intima-media thickness, *Corr* correlation coefficient

^^^ *p* < 0.05

### Associations between homocysteine metabolism related SNPs and biochemical indexes

rs1801133 T allele was associated with elevated plasma Hcy levels (*p* = 0.012). rs1532268 A allele was associated with elevated plasma VB6 levels (*p* = 0.010). rs2851391 T allele was associated with decreased plasma VB12 levels (*p* = 0.036) (Table 3).

**Table 3:** Associations between homocysteine metabolism related SNPs and biochemical indexes

SNP	Geno-type	Hcy		VB6		VB12		FA		PAI-1	
		Mean ± SD	p*	Mean ± SD	p*	Mean ± SD	p*	Mean ± SD	p*	Mean ± SD	p*
rs1801133	CC	2.53 ± 0.50	0.012^^^	9.13 ± 0.56	0.482	6.33 ± 0.59	0.906	8.87 ± 0.52	0.304	11.44 ± 0.91	0.524
	CT	2.56 ± 0.40		9.04 ± 0.58		6.36 ± 0.64		8.86 ± 0.53		11.66 ± 0.58	
	TT	2.77 ± 0.46		9.27 ± 0.58		6.55 ± 0.51		8.86 ± 0.47		11.58 ± 0.58	
rs1805087	AA	2.64 ± 0.45	0.947	9.15 ± 0.58	0.082	6.37 ± 0.62	0.877	8.88 ± 0.51	0.068	11.57 ± 0.69	0.382
	AG	2.71 ± 0.45		8.88 ± 0.47		6.40 ± 0.52		8.70 ± 0.48		11.44 ± 1.03	
	GG	2.78 ± 0.76		8.86 ± 0.31		6.14 ± 0.29		8.63 ± 0.54		11.63 ± 0.78	
rs16879248	TT	2.62 ± 0.45	0.391	9.06 ± 0.55	0.409	6.34 ± 0.58	0.886	8.88 ± 0.50	0.094	11.63 ± 0.67	0.898
	TC	2.64 ± 0.51		9.05 ± 0.62		6.33 ± 0.61		8.76 ± 0.53		11.55 ± 0.83	
	CC	2.83 ± 0.52		9.51 ± 0.89		6.93 ± 0.69		8.92 ± 0.53		11.92 ± 0.34	
rs1532268	GG	2.64 ± 0.48	0.299	9.07 ± 0.57	0.010^^^	6.39 ± 0.61	0.364	8.86 ± 0.52	0.083	11.59 ± 0.75	0.786
	GA	2.63 ± 0.42		9.08 ± 0.61		6.38 ± 0.52		8.75 ± 0.49		11.52 ± 0.78	
	AA	2.58 ± 0.24		9.15 ± 0.53		6.11 ± 0.84		9.12 ± 0.49		11.42 ± 1.05	
rs10037045	AA	2.64 ± 0.42	0.535	9.11 ± 0.58	0.530	6.37 ± 0.62	0.639	8.85 ± 0.51	0.108	11.59 ± 0.75	0.242
	AT	2.69 ± 0.52		9.10 ± 0.56		6.42 ± 0.57		8.90 ± 0.51		11.54 ± 0.76	
	TT	2.68 ± 0.45		9.07 ± 0.67		6.37 ± 0.61		8.82 ± 0.61		11.35 ± 0.62	
rs3733890	GG	2.63 ± 0.43	0.573	9.09 ± 0.59	0.181	6.37 ± 0.61	0.692	8.83 ± 0.51	0.074	11.56 ± 0.76	0.315
	GA	2.66 ± 0.52		9.03 ± 0.55		6.36 ± 0.58		8.85 ± 0.52		11.61 ± 0.77	
	AA	2.58 ± 0.49		9.13 ± 0.64		6.46 ± 0.59		8.84 ± 0.56		11.47 ± 0.68	
rs11868708	TT	2.64 ± 0.47	0.543	9.11 ± 0.58	0.426	6.33 ± 0.59	0.158	8.87 ± 0.47	0.917	11.59 ± 0.81	0.565
	TC	2.64 ± 0.47		9.03 ± 0.56		6.38 ± 0.57		8.83 ± 0.56		11.63 ± 0.64	
	CC	2.58 ± 0.45		9.05 ± 0.62		6.43 ± 0.69		8.84 ± 0.53		11.76 ± 0.30	
rs2851391	CC	2.67 ± 0.47	0.426	9.14 ± 0.62	0.869	6.43 ± 0.61	0.036^^^	8.88 ± 0.47	0.419	11.52 ± 0.76	0.284
	CT	2.63 ± 0.43		9.09 ± 0.53		6.35 ± 0.60		8.84 ± 0.53		11.59 ± 0.76	
	TT	2.75 ± 0.46		9.05 ± 0.51		6.30 ± 0.61		8.90 ± 0.66		11.55 ± 0.61	
rs234709	CC	2.64 ± 0.46	0.095	9.07 ± 0.59	0.273	6.42 ± 0.59	0.883	8.89 ± 0.51	0.053	11.56 ± 0.79	0.283
	CT	2.54 ± 0.43		9.11 ± 0.59		6.35 ± 0.59		8.83 ± 0.52		11.72 ± 0.64	
	TT	2.88 ± 0.63		8.97 ± 0.60		5.9 ± 0.39		8.83 ± 0.69		11.46 ± 0.44	

*p for Family-based association test analysis under additive model, adjusting for age, sex, smoking, BMI, diabetes, SBP, TG, LDL-C, ^^^ *p* < 0.05

### Associations between homocysteine metabolism related SNPs and carotid intima-media thickness in family-based association test analysis

When analyzing associations between 9 SNPs and CIMT indexes using family-based association test analysis (FBAT), we found that CBS rs2851391 was significantly associated with CCA-IMT (*p* = 0.021) and CIMT (*p* = 0.019), MTRR rs1532268 was significantly associated with CCA-IMT (*p* = 0.040). No significant association was found between Bif-IMT and homocysteine metabolism related SNPs (Table 4).

**Table 4:** Associations between homocysteine metabolism related SNPs and CIMT indexes in family-based association test analysis

Gene	SNP	Location	Alleles	MAF	HWE *p* value	Fam^#^	*p* for CCA-IMT	*p* for Bif-IMT	*p* for CIMT
MTHFR	rs1801133	1:11796321	C/T	T(0.379)	0.573	93	0.722	0.282	0.464
MTR	rs1805087	1:236885200	A/G	G(0.071)	0.765	22	0.839	0.873	0.875
MTRR	rs16879248	5:7864419	T/C	C(0.146)	0.784	47	0.855	0.970	0.903
MTRR	rs1532268	5:7878066	G/A	A(0.134)	0.866	46	0.040^^^	0.453	0.133
BHMT	rs10037045	5:79126747	A/T	T(0.223)	0.246	68	0.111	0.095	0.066
BHMT	rs3733890	5:79126136	G/A	A(0.254)	0.819	76	0.078	0.102	0.056
SHMT1	rs11868708	17:18341299	T/C	C(0.295)	0.289	77	0.130	0.829	0.287
CBS	rs2851391	21:43067294	C/T	T(0.295)	0.836	78	0.021^^^	0.095	0.019^^^
CBS	rs234709	21:43066854	C/T	T(0.112)	0.651	38	0.562	0.314	0.388

Family-based association test analysis was under additive model, and adjusted for age, sex, smoking, BMI, diabetes, SBP, TG, LDL-C

*HWE* Hardy–Weinberg equilibrium, *MAF* minor allele frequency, described as minor allele (minor allele frequency)

^#^Fam: Numbers of informative families, ^^^ *p* < 0.05

### Associations between homocysteine metabolism related SNPs and carotid intima-media thickness in multi-level mixed-effects regression model

To replicate FBAT result, we further analyzed associations between 9 SNPs and CIMT indexes using multi-level mixed-effects regression model. SHMT1 rs11868708 was associated with CCA-IMT (Coef = 0.007, se = 0.008, raw *p* = 0.013) and CIMT (Coef = 0.011, se = 0.005, raw *p* = 0.024). CBS rs2851391was associated with CCA-IMT (Coef = 0.032, se = 0.009, raw *p* < 0.001), Bif-IMT (Coef = 0.025, se = 0.011, raw *p* = 0.019) and CIMT (Coef = 0.023, se = 0.010, raw *p* = 0.018).

After Bonferoni correction, CBS rs2851391 remained significantly associated with CCA-IMT (α = 0.0056). Compared with individuals with no T alleles on rs2851391, every extra T allele can result in 0.032 millimeters (mm) thickening of CIMT. No associations between SNPs and Bif-IMT or CIMT reached Bonferoni corrected α threshold (Table 5).

**Table 5:** Associations between homocysteine metabolism related SNPs and CIMT indexes in multi-level mixed-effects regression model

Gene	SNP	CCA-IMT			Bif-IMT			CIMT		
		Index changes per risk allele^#^	SE	Raw p*	Index changes per risk allele^#^	SE	Raw p*	Index changes per risk allele^#^	SE	Raw p*
MTHFR	rs1801133	0.007	0.008	0.374	0.008	0.010	0.434	0.007	0.009	0.438
MTR	rs1805087	0.019	0.015	0.206	0.007	0.019	0.714	0.016	0.017	0.327
MTRR	rs16879248	0.002	0.011	0.831	0.005	0.013	0.725	0.001	0.011	0.996
MTRR	rs1532268	0.009	0.011	0.417	−0.001	0.013	0.959	0.004	0.012	0.723
BHMT	rs10037045	0.002	0.009	0.835	0.007	0.011	0.493	0.008	0.010	0.425
BHMT	rs3733890	−0.007	0.005	0.155	−0.002	0.006	0.769	−0.006	0.005	0.215
SHMT1	rs11868708	0.007	0.008	0.013^^^	0.011	0.010	0.277	0.011	0.005	0.024^^^
CBS	rs2851391	0.032	0.009	<0.001^^^^	0.025	0.011	0.019^^^	0.023	0.010	0.018^^^
CBS	rs234709	0.005	0.007	0.503	−0.003	0.009	0.716	0.001	0.007	0.907

*Coef* coefficients, *SE* standard error

*Adjusting for age, sex, smoking, BMI, diabetes, SBP, TG, LDL-C

^#^Coefficients for SNPs in mixed models, ^^^ *p* < 0.05, ^^^^ *p* < 0.01

### Gene–gene interactions between CBS rs2851391 and other homocysteine related SNPs on carotid intima-media thickness

Gene–gene interactive effects were analyzed under dominant genetic model in multi-level mixed-effects regression model. An interaction was found between CBS rs2851391 and BHMT rs10037045 for CCA-IMT (*p* = 0.011). The combination of CBS rs2851391 CT/TT and BHMT rs10037045 AT/TT genotypes can increase CCA-IMT by 0.080 mm. Interactions were found between CBS rs2851391 and MTR rs1805087 for CCA-IMT (*p* = 0.007) and CIMT (*p* = 0.022). The combination of CBS rs2851391 CT/TT and MTR rs1805087 AG/GG genotypes can increase CCA-IMT by 0.101 mm and CIMT by 0.089 mm (Table 6).

**Table 6:** Gene–gene interactive effects of homocysteine related SNPs on CIMT indexes

SNP	Model	CCA-IMT	Bif-IMT	CIMT
SNP1: CBS rs2851391 × SNP2: BHMT rs10037045	Index changes if risk allele of SNP1 exists^a^	0.051	0.042	0.043
	Index changes if risk allele of SNP2 exists^b^	−0.023	−0.003	−0.013
	Index changes if risk alleles of both SNPs coexist^c^	0.080	0.061	0.075
	*p* for interaction	0.011^^^	0.402	0.052
SNP1: CBS rs2851391 × SNP2:MTR rs1805087	Index changes if risk allele of SNP1 exists^a^	0.042	0.039	0.035
	Index changes if risk allele of SNP2 exists^b^	−0.021	−0.011	−0.020
	Index changes if risk alleles of both SNPs coexist^c^	0.101	0.073	0.089
	*p* for interaction	0.007^^^^	0.227	0.022^^^

Adjusting for age, sex, smoking, BMI, diabetes, SBP, TG, LDL-C

^a^Coefficient for SNP1 in full mixed model

^b^Coefficient for SNP2 in full mixed model

^c^Sum of coefficients for SNP1, SNP2 and interaction term in full mixed model

^^^ *p* < 0.05, ^^^^ *p* < 0.01

## Discussion

The accessibility and reproducibility of CIMT measurement varies significantly at different carotid artery segments [over 90% for CCA, 60–80% for bifurcation and 30–50% for internal carotid artery (ICA)] [17]. In the present study, we recorded images of bilateral ICA. However, ICA IMT can only be clearly measured in 38.6% participants. Thus it was not involved in further statistical analysis. The associations between CIMT at various segments and cardiovascular diseases are also different. Thickening of CCA-IMT may reflect the pathological changes of medial hypertrophy as a result of smooth muscle cell hyperplasia or fibrocellular hypertrophy rather than the formation of atherosclerotic plaque [18]. Epidemiological studies suggested CCA-IMT as a strong predictor for cardiovascular events. It was associated with myocardial infarction and stroke [2, 19], which was later proved by a meta-analysis [20]. Carotid bifurcation is a region where blood in carotid artery flows into two vessels of unequal sizes. The consequent sudden changes of hemodynamic factors, such as variations in flow velocity, shear stress and turbulence, make carotid bifurcation an atherosclerosis-prone location [21, 22]. Ebrahim et al. reported that the associations between CCA-IMT and stroke were independent of carotid plaques, whereas associations between Bif-IMT and ischemic heart disease can be largely explained by the presence of carotid plaques [23].

B-group vitamins are essential coenzymes in Hcy metabolism. Evidences indicated that increased CIMT was associated with higher Hcy and lower folic acid levels [24]. Although we observed no associations between Hcy levels and CIMT indexes, significant negative associations between FA, VB6, VB12 and CIMT indexes were found in the present study. B vitamins (FA, VB6, VB12) can prevent atherogenesis through their antioxidant properties and their ability to lower plasma homocysteine levels [25, 26]. However, the effect of folic acid and vitamin B supplementation on CIMT progression was still controversial [27].

Plasminogen activator inhibitor-1, a component of the plasminogen/plasmin system, was considered as a link between inflammation, insulin resistance and vascular risks, such as atherogenesis and atherothrombosis [28, 29]. PAI-1 was suggested to be a predictor for cardiovascular disease [30]. However, associations between PAI-1 and CIMT were not consistent in different studies [31, 32]. We tested PAI-1 in the present study but found no associations between PAI-1 and CIMT indexes.

MTHFR 677 C>T (rs1801133) is the most frequently studied functional genetic polymorphism proved to be independently associated with folate, homocysteine, and vascular endothelial dysfunction [33–35]. However, association between MTHFR 677 C>T genotype and CIMT in different ethnic populations remains controversial [5, 36–38]. There is also no evidence that MTHFR 677 C>T has an effect on coronary artery disease [39]. In the present study, we observed an association between rs1801133 T allele and elevated plasma Hcy levels, but found null association between MTHFR 677 C>T and CIMT indexes. These results indicated that the thickening of CIMT might be a pathological process resulting from complex metabolic disorders rather than isolated hyperhomocysteinemia.

SHMT1 rs11868708 risk allele conferred a 1.46-fold risk of ischemic stroke in Singaporean Chinese. The total number of risk alleles on MTRR rs16879248, SHMT1 rs11868708, TCN2 rs1173570 showed a cumulative effect on ischemic stroke [40]. These three SNPs were genotyped in the present study. The association between SHMT1 rs11868708 and CCA-IMT (raw *p* = 0.013) didn’t reach Bonferoni corrected α threshold. More studies are needed to investigate the role SHMT1 rs11868708 plays in atherogenesis and subsequent cardiovascular diseases.

Cystathionine-β-synthase (CBS) transforms homocysteine to cystathionine in the assistance of vitamin B6. CBS 68-bp insertion (844ins68) carriers have been reported to lower plasma Hcy levels [41]. Rs2851391 located in the intron of CBS. A GWAS meta-analysis showed that CBS rs234709 and CBS rs2851391 were associated with Hcy concentrations [9]. Zinck et al. discussed that rs2851391 variants might reduce the activity of CBS, and thus was positively associated with homocysteine levels [42]. Rs2851391 genetic polymorphism is associated with neural tube defects. Its risk genotype has a 2.0-fold risk of spina bifida [43]. Hsu et al. indicated that rs2851391 polymorphisms were associated with changes in postmethionine load Hcy levels, but it was not associated with recurrent stroke risk [44]. In the present study, we identified CBS rs2851391 as a risk factor for CCA-IMT but not Bif-IMT. This might result from the different pathology for CCA-IMT and Bif-IMT thickening. CBS rs2851391 T allele showed a marginal association with decreased plasma VB12 level, which might be a possible explanation for its association with CCA-IMT.

Methionine synthase (MTR) catalyzes the remethylation of homocysteine to methionine [45]. MTR rs1805087 (A2756G) is a well-studied genetic polymorphism. Laraqui et al. defined Hcy level ≥15 μmol/l as hyperhomocyteinemia (HHcy), and found that MTR rs1805087 (A2756G) G allele contributed a 2.0-fold risk for HHcy [46]. The G allele can also increase DNA methylation level [47]. Masud et al. reported significant associations of rs1805087 with coronary artery disease under additive and dominant models [48].

Gene–gene interactions between homocysteine related SNPs are studied before. Many studies focus on interactions between MTR rs1805087 and genetic polymorphisms of other homocysteine metabolism related genes. The combination of MTR A2756G AG/GG, MTHFR 677 CT/TT and MTHFR 1298 AC/AA genotypes contributes significantly to extremely high Hcy plasma levels [49]. Interaction between MTR A2756G and MTRR A66G may lead to an increase of infants’ neural tube defects risks [50]. An existence of gene–gene interaction between rs1801133, rs662 and rs1805087 was reported for coronary artery disease [48]. MTR rs1805087 is located in the AdoMet-binding region, this polymorphism results in a nonconservative substitution of aspartic acid residue by glycine residue, which could influence the secondary structure and function of the protein [51]. In the present study, we observed an association of distinct CBS rs2851391/MTR rs1805087 genotype combination with elevated CCA-IMT and CIMT, which might be an indication of interaction between Hcy remethylation pathway and transsulfuration pathway. BHMT rs10037045 is reported to be a risk factor for ischemic stroke [52]. This SNP is not well studied before. More researches are needed to understand the role BHMT rs10037045 plays in atherogenesis and cardiovascular diseases, and its interaction with CBS rs2851391.

Compared with parent-offspring trios study, sib pair study is more suitable for late-onset diseases, since the parents’ phenotypic and genotypic traits may not be available at the time offspring develops the certain disease. What’s more, sib pairs are more closely matched for age and environmental factors than other relative pairs [53]. The shared genetic background for siblings from same pedigree prevented the problem of population stratification, a cause for false positive associations in population based case-control studies. As a consequent, the power of sib pair study is relatively low [54].

The present study has some limitations. Firstly, we only selected 9 SNPs located on homocysteine related genes using candidate gene approach. These 9 SNPs are previously reported to be associated with Hcy levels or cardiovascular diseases, or to have interactions with risk SNPs for cardiovascular diseases. Thus, the aim of the present family-based sib pair study is to verify positive results found in population based studies, rather than to identify new risk SNPs. Secondly, the present study is a cross-sectional study. The causality of the associations is thus compromised. However, since human genetic profile is unchangeable during life time, the genetic risk factors can be considered as being prior to disease occurrence. On the other hand, this can’t deny the need for longitudinal studies to elucidate the relationships between homocysteine related SNPs and CIMT or CIMT changes. Thirdly, CIMT, especially CCA-IMT, is a good surrogate for cardiovascular events. The associations between risk SNPs and CIMT indexes suggest a possible influence of these SNPs on cardiovascular diseases. However, it is of greater value to directly elaborate the associations between SNPs and individual diseases. FISSIC study is an ongoing family-based genetic epidemiological study. Our next goal is to investigate the role genetic risk factors play in ischemic stroke, coronary artery disease and type 2 diabetes.

## Conclusions

Significant associations are found between Hcy related genetic polymorphisms, biochemical indexes and carotid intema-media thickness. There are complex interactions between Hcy related genetic polymorphisms.
